# Improving health system responses when patients are harmed: a protocol for a multistage mixed-methods study

**DOI:** 10.1136/bmjopen-2024-085854

**Published:** 2024-07-05

**Authors:** Peter D Hibbert, Louise Raggett, Charlotte J Molloy, Johanna Westbrook, Farah Magrabi, Virginia Mumford, Robyn Clay-Williams, Raghu Lingam, Paul M Salmon, Sandy Middleton, Mike Roberts, Patricia Bradd, Steven Bowden, Kathleen Ryan, Mark Zacka, Kirstine Sketcher-Baker, Andy Phillips, Lanii Birks, Dinesh K Arya, Catherine Trevorrow, Suchit Handa, Girish Swaminathan, Andrew Carson-Stevens, Siri Wiig, Carl de Wet, Elizabeth E Austin, Brona Nic Giolla Easpaig, Ying Wang, Gaston Arnolda, Gregory M Peterson, Jeffrey Braithwaite

**Affiliations:** 1 Australian Institute of Health Innovation, Faculty of Medicine and Health Sciences, Macquarie University, Sydney, New South Wales, Australia; 2 IIMPACT in Health, Allied Health and Human Performance, University of South Australia, Adelaide, South Australia, Australia; 3 School of Women's and Children's Health, University of New South Wales, Sydney, New South Wales, Australia; 4 Centre for Human Factors and Sociotechnical Systems, University of the Sunshine Coast, Sippy Downs, Queensland, Australia; 5 Nursing Research Institute, St Vincent's Health Network Sydney and Australian Catholic University, St Vincent's Hospital Melbourne Pty Ltd, Fitzroy, Victoria, Australia; 6 School of Nursing, Midwifery and Paramedicine, Australian Catholic University, Fitzroy, Victoria, Australia; 7 Safer Care Victoria, Melbourne, Victoria, Australia; 8 Clinical Excellence Commission, St Leonards, New South Wales, Australia; 9 Mid North Coast Local Health District, Port Macquarie, New South Wales, Australia; 10 Northern Sydney Local Health District, St Leonards, New South Wales, Australia; 11 Clinical Excellence Queensland, Health Innovation and Research Branch, Queensland Health, Brisbane, Queensland, Australia; 12 ACT Health, Canberra, Australian Capital Territory, Australia; 13 Australian Commission on Safety and Quality in Healthcare, Sydney, New South Wales, Australia; 14 PRIME Centre Wales & Division of Population Medicine, Cardiff University, Cardiff, UK; 15 Centre for Resilience in Healthcare (SHARE), Faculty of Health Sciences, University of Stavanger, Stavanger, Norway; 16 South West Hospital and Health Service, Roma, Queensland, Australia; 17 School of Nursing, Charles Darwin University, Darwin, Northern Territory, Australia; 18 School of Pharmacy and Pharmacology, University of Tasmania, Hobart, Tasmania, Australia

**Keywords:** Safety, QUALITATIVE RESEARCH, Quality Improvement, Quality in health care, Clinical governance

## Abstract

**Introduction:**

At least 10% of hospital admissions in high-income countries, including Australia, are associated with patient safety incidents, which contribute to patient harm (‘adverse events’). When a patient is seriously harmed, an investigation or review is undertaken to reduce the risk of further incidents occurring. Despite 20 years of investigations into adverse events in healthcare, few evaluations provide evidence of their quality and effectiveness in reducing preventable harm.

This study aims to develop consistent, informed and robust best practice guidance, at state and national levels, that will improve the response, learning and health system improvements arising from adverse events.

**Methods and analysis:**

The setting will be healthcare organisations in Australian public health systems in the states of New South Wales, Queensland, Victoria and the Australian Capital Territory. We will apply a multistage mixed-methods research design with evaluation and in-situ feasibility testing. This will include literature reviews (stage 1), an assessment of the quality of 300 adverse event investigation reports from participating hospitals (stage 2), and a policy/procedure document review from participating hospitals (stage 3) as well as focus groups and interviews on perspectives and experiences of investigations with healthcare staff and consumers (stage 4). After triangulating results from stages 1–4, we will then codesign tools and guidance for the conduct of investigations with staff and consumers (stage 5) and conduct feasibility testing on the guidance (stage 6). Participants will include healthcare safety systems policymakers and staff (n=120–255) who commission, undertake or review investigations and consumers (n=20–32) who have been impacted by adverse events.

**Ethics and dissemination:**

Ethics approval has been granted by the Northern Sydney Local Health District Human Research Ethics Committee (2023/ETH02007 and 2023/ETH02341).

The research findings will be incorporated into best practice guidance, published in international and national journals and disseminated through conferences.

**STRENGTHS AND LIMITATIONS OF THIS STUDY**

- Four-year multistage mixed-methods study.
- Engagement with a diverse group of consumers, health service staff and policymakers.
- Designed to account for complex adaptive sociotechnical healthcare systems.
- Development of best practice guidelines using in situ feasibility testing.
- Direct measurement of the quality of investigations is limited to assessing artefacts.

## Introduction

The WHO, governments, research and clinical communities recognise patient safety as a public health issue with significant societal and economic burden.^1–7^ According to WHO, around 1 in every 10 patients suffer a patient safety incident in healthcare, which contributes to them being harmed (‘adverse events’), and as many as 4 in 10 patients are harmed in primary and ambulatory settings, with more than 3 million deaths annually due to adverse events.^8^ Over 50% of adverse events are preventable.^9^ Patient harm potentially reduces global economic growth by 0.7% a year.^10^ On a global scale, the indirect cost of adverse events amounts to trillions of US dollars each year.^10 11^ The magnitude of the issue in Australia, measured by prevalence of patient harm (at least 10% of hospital admissions^2 12^ or the associated cost to the health system (AUD$4.1 billion or 8.9% of Australian hospital expenditure per annum),^4^ is similarly disturbing. There is also frustration at the slow pace of making gains in reducing harm to patients since the turn of the century.^13–16^

When a patient is seriously harmed by an adverse event (such as contributing to death or permanent harm^17 18^), an investigation (in some health services, this is called a review) is undertaken to determine what happened and why and to develop interventions that aim to reduce further patients being harmed. Over 1000 adverse event investigations are undertaken each year in Australian public health systems.^19^ There is currently a lack of Australian data available on the cost of investigations, but extrapolating figures from the UK’s National Health Service (NHS)^2^ the opportunity cost in staff time of managing incidents in the state of New South Wales (NSW) alone is estimated at AUS$123M annually.

Both internationally and within Australia concerns have been raised regarding the quality and effectiveness of adverse event investigations.^2 15 20–23^ Despite 20 years of investigations into adverse events in healthcare, few evaluations provide evidence of their effectiveness in reducing preventable harm. A systematic review of the effectiveness of investigations found that only 2 of 21 studies showed a subsequent reduction in harm.^20^ One study of 227 adverse event investigations from the Victorian public health system found that only 8% of recommendations were likely to be effective and sustainably implemented.^2 21^ There have been similar findings from NSW^24^ and, internationally, from New York.^23^ There is also little understanding of new investigation models in healthcare, or the most effective systems to implement recommendations after an investigation^15 22^ or their impact in reducing preventable harm.

An investigation should take into account the perspectives of patients and their families and carers.^25^ They may have insights into what and why happened in relation to the adverse event and have particular questions that they think are important for the investigation to tackle. As well as the investigation, an organisational response by a health service to adverse events may involve psychological, medical and financial support to the affected patient and their family and open disclosure.^26^ Whether the health service’s overall response, including the investigation, adequately listens to and supports patients and family can compound their psychological impact, resulting from the initial adverse event.^27^

Our research will develop and test evidenced-based guidelines for healthcare organisations to improve and measure their response, analysis and learning from adverse events, focused particularly on serious ones, thereby aiming to reduce preventable harm to patients. This study aims to develop consistent, informed and robust best practice guidance, at state and national levels, which will improve the response, learning and health systems improvements arising from adverse events. The study will apply multistage mixed-methods, together with evaluation and in situ feasibility testing, to achieve its aims.

## Methods and analysis

The study has three research questions:

1. How should we decide what to investigate and to what level, to maximise learning to improve patient safety—making the best use of the limited resources available?
2. How can we ensure investigations are of sufficient quality to identify contributing factors and identify effective system improvements?
3. Why do the recommended actions from investigations sometimes fail to generate systematic, sustainable improvements to patient safety—and how could we improve this in the future?

**Figure 1:**
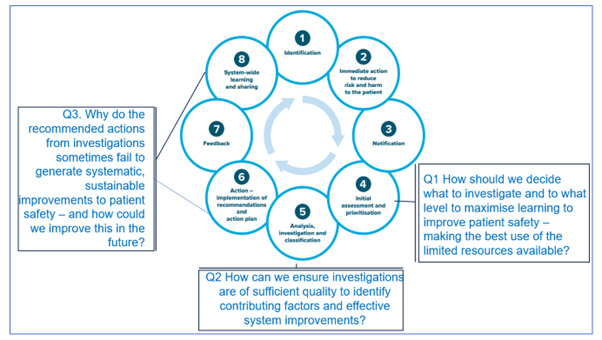
Research questions related to the Australian Commission on Safety and Quality in Health—Incident Management Guide phases of incident management. Adapted from the Phases of Incident Management, Australian Commission on Safety and Quality in Health Care—Incident Management Guide 2021 Page 5.^62^ Permission received from the Australian Commission on Safety and Quality in Health Care to use this figure on 11 January 2024.

The research questions relate to different phases of the incident management cycle, as shown in figure 1.

### Partners and healthcare systems involved

Four public state/territory health systems and associated care quality agencies in Australia (NSW via the Clinical Excellence Commission; Victoria via Safer Care Victoria; Queensland via Clinical Excellence Queensland; and the Australian Capital Territory (ACT) via ACT Health), together with the Australian Commission on Safety and Quality in Healthcare, have partnered with the Australian Institute of Health Innovation at Macquarie University, in a National Health and Medical Research Council (NHMRC) Partnership Grant (number 2017219), to undertake this research. The University of New South Wales, University of the Sunshine Coast, and Australian Catholic University are partners in the research. Collectively, the participating states and territory comprise 79% of Australia’s population and cover 79% of hospital admissions (table 1).^28–30^

**Table 1:** Population, number of hospital admissions and emergency department presentations by research jurisdictional partner and Australia, 2021–2022^28–30^

Partner states/territory Australia	Population (% of Australia)^28^	Number of hospital admissions (% of Australia)^29^	Number of emergency department presentations (% of Australia)^30^
Australian Capital Territory	455 657 (1.8)	121 079 (1.8)	143 693 (1.6)
New South Wales	8 137 688 (31.4)	1 783 103 (26.1)	3 012 992 (34.3)
Queensland	5 294 256 (20.4)	1 720 372 (25.2)	1 867 860 (21.3)
Victoria	6 604 868 (25.5)	1 797 400 (26.3)	1 856 312 (21.1)
Partner jurisdiction totals	20 492 469 (79.1)	5 421 954 (79.4)	6 880 857 (78.3)
Australia	25 909 850	6 827 706	8 789 877

All partners in the research recognise that effective consumer partnerships are essential for improving healthcare outcomes and driving continuous improvement.^8 31–34^ Empowering consumers to partner in the design of safety processes, including guidance for investigations, enables better understanding of their needs, concerns and values. Our research governance that includes consumers, and our codesign approach (see stages 4–6), both aim to incorporate these consumer needs, concerns and values into our study across all three research questions.

**Figure 2:**
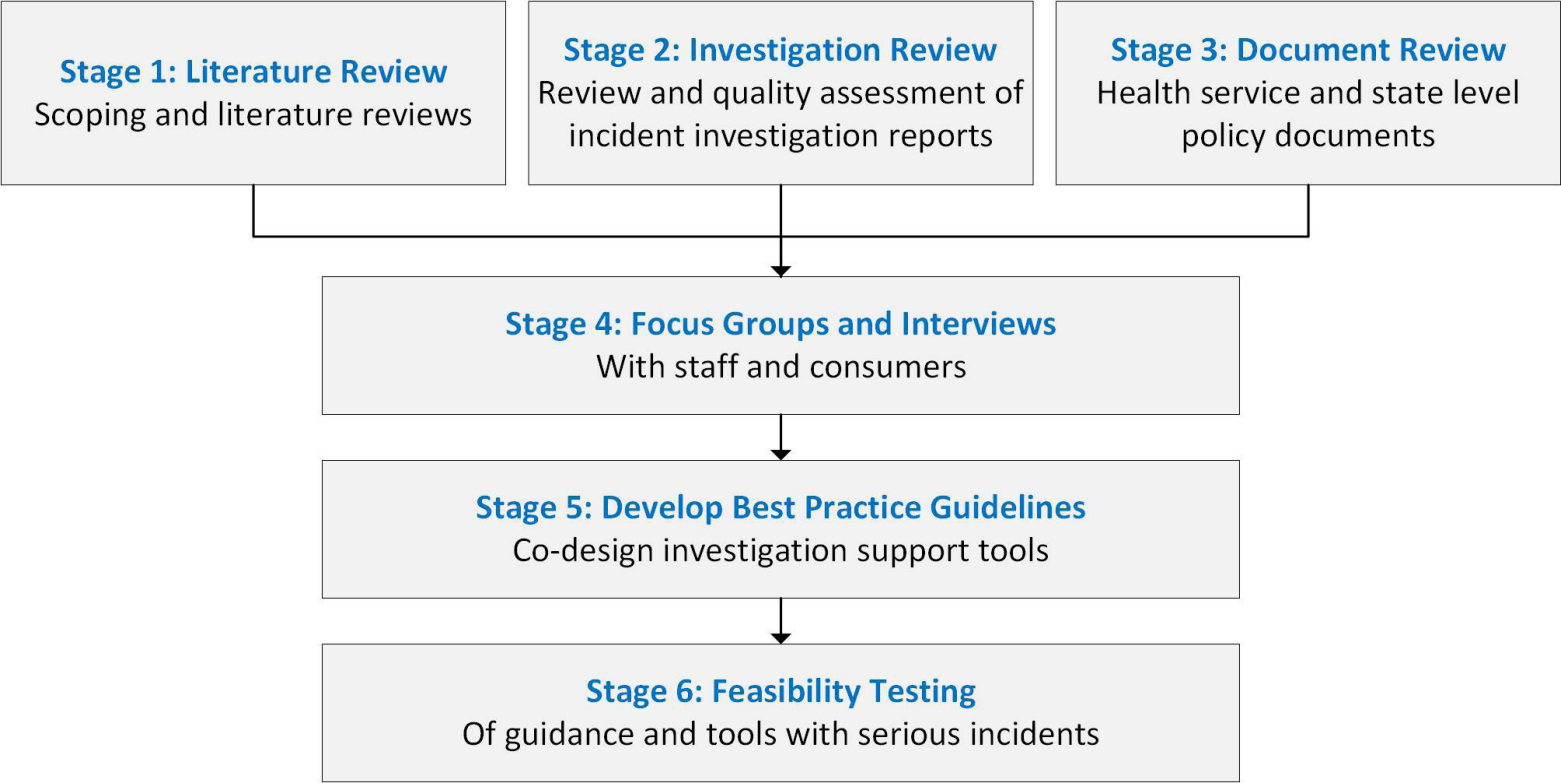
Project stages for improving the health system’s response when patients are harmed.

When healthcare organisations respond to adverse events, including undertaking investigations, they do so within complex adaptive sociotechnical systems.^35^ Local team and organisational cultural maturity, commitment to safety, historical attitude to blame, governance structures, resources and demand pressures and political and social factors are likely to impact the effectiveness of the response as much as the composition and skills of the investigation team and technical attention to the investigation method. Therefore, this research into adverse event investigations has been designed to take into account complex, multicontextual factors. The research will be conducted over six stages as shown in figure 2 and will be conducted between 2024 and 2026.

### Stage 1—literature review

Two literature reviews will be conducted, which will inform the interviews and focus groups with stakeholders (stage 4) and development of best practice guidance (stage 5).

First, an international grey literature review will examine the types of adverse events and in what circumstances they should be investigated. This review will examine published material from safety policy agencies investigating adverse events. Websites and documents in national or state/provincial agencies in primarily high-income countries will be searched. This will include, but not be limited to, NHS England/Scotland/Wales, England’s Health Services Safety Investigations Body (HSSIB), Healthcare Excellence Canada, the Norwegian Healthcare Investigation Board and the Safety Investigation Authority of Finland. The aim is to understand how such agencies decide what to investigate and what level of investigation is most appropriate, including any decision-making criteria or prioritisation tools used. These will include criteria related to consumer needs. The decision-making criteria will be inductively categorised and descriptively analysed. The outcome of the literature review will be an analysis of frequently used decision-making criteria to decide when to undertake an investigation. These criteria will be discussed during stage 4 focus groups and interviews.

Second, a scoping review will be conducted on the contemporary use of systems frameworks.^36^ The use of systems frameworks is becoming more common to review adverse events and analyse health system quality. This scoping review will include research and narrative reports on the application of systems frameworks in any healthcare setting. The focus will be to identify the uses and applications of systems frameworks, the methods used to operationalise them, the healthcare processes examined and the key findings. The review will consider peer-reviewed journal articles in CINAHL, MEDLINE, Embase and PsycINFO, from any country, published in English, with no limits on the publication year. A search of the grey literature will also be conducted. It is anticipated that the scoping review will provide further insight into how systems frameworks can be operationalised in healthcare systems to strengthen patient safety.

### Stage 2—review the quality of investigations

#### Purpose

We will undertake a qualitative deductive content analysis^37^ of 300 patient safety investigation reports. The aim is to assess their quality using four existing assessment frameworks and determine the incident types associated with the investigation reports. Stage 2 is aligned with research question 2.

#### Setting

The study setting is health services within the three of the four partner jurisdictions—NSW, Queensland and Victoria. Legislation in the ACT precludes sharing of the investigation reports within a research environment. The investigation reports will be accessed via the partner organisations.

#### Data sources

Health services undertake investigations which inform reports (‘investigation reports’) when there is an adverse event. The inclusion criteria will be investigation reports undertaken on adverse events designated as the highest level of severity within each jurisdiction. We will analyse 100 each from NSW, Victoria and Queensland. A sample of 100 at state level will allow CIs of+/−10% points around a point estimate of 50%.

Data in the investigation reports include details such as the incident type, investigation type, date, state, health service and location within the health service. The main body of the report is in a narrative form outlining the nature of the adverse event, the contributing factors and the risk reduction action plans/recommendations, which propose actions to reduce the risk of adverse event recurrence.

#### Sampling

We will request a list, from the partners, of all investigation reports completed within the three states during 2022 and 2023. The lists will contain several fields to facilitate the selection of a representative sample of investigation reports from each jurisdiction. These fields include:

- Record/investigation number (to identify and select those to request for analysis).
- Whether or not the investigation was protected by legislation.
- The investigation method (eg, root cause analysis, London Protocol^38^).
- The health service location where the adverse event took place (to identify the size and rurality of the facility).
- Medical specialty related to the adverse event.
- Whether the adverse event was a notifiable and/or sentinel event.

On receipt of the list, the research team will stratify the investigation reports into five categories specified above (protected by legislation, investigation method, health service location, specialty, notifiable/sentinel event) and select randomly until we have a representative sample (100 Investigation Reports for each state) based on these variables. We intend to oversample lesser used investigation types and smaller health facilities to increase our confidence in the results of our findings in these subsamples; the overall result will be appropriately weighted to account for over-sampling.

Once the investigation reports being included in the sample are identified, we will formally request the selected reports to be transferred to a Macquarie University secure SharePoint portal. The 300 investigation reports will then be allocated a unique study ID. Any small cell sizes with variables or characteristics with the potential to be reidentified will be aggregated into broader categories such that the reported data are non-identifiable.

#### Data Collection

The incident type related to the investigation will be classified according to the high-level classes of a modified version of the WHO’s International Classification for Patient Safety.^39^ Demographic information related to the patient (eg, age (recorded in age bands, eg, 60–69 years) and gender) will be extracted from the reports.

The quality assessment review, using a deductive content analysis, involves reading and coding the investigation reports and extracting information from them guided by four quality assessment frameworks:

1. The Healthcare Inspectorate tool from the Netherlands^40^ assesses adherence to guidance for patient safety investigation methods (eg, investigation process and team, adverse event reconstruction, analysis, conclusion, recommendations, aftercare and board responses).

2. The US Department of Veteran Affairs’ recommendations effectiveness scale^41^ assesses recommendations’ relative ‘strength’ according to their type, effectiveness and sustainability. According to the criteria, effective reports should have clear causal statements and strong actions. Strong actions are most likely to be system-based according to human factors engineering principles.^42^

3. Accimaps and SEIPS.^36 43^ The SEIPS framework identifies work system descriptors that might have contributed to an adverse event, also known as ‘contributing factors’. SEIPS categorises these into external influences, organisation of work factors, task factors, person factors, tools and technology and the physical environment. These apply at different levels of a healthcare system. Accimaps is a system-based incident analysis technique. An output of an Accimap is a layered causal diagram, which shows the relationships of contributing factors across the different levels of a system. By combining the SEIPS categories with simplified Accimaps levels, the assessment can identify the breadth of healthcare system levels considered during each investigation.

4. The Learning Response Review and Improvement Tool from NHS Scotland and HSSIB.^25^ The tool rates the investigation according to criteria considered good practice for systems investigations to identify areas for improvement and to act as a quality assurance process for monitoring and evaluating the standard of organisational learning response reports. The tool includes criteria related to whether both patients (and their families and carers) and staff have been actively listened to and emotionally supported where required.^25^

The data items within each of the four quality assessment frameworks against which the text in the investigation reports will be coded are shown in online supplemental material 1. The research team will develop data collection tools in a Microsoft Access database to record the quality assessment framework data items. The databases will be securely stored on the Macquarie University SharePoint platform.

10.1136/bmjopen-2024-085854.supp1 Supplementary data

Experienced safety science researchers will undertake the coding. They will independently code the first five investigations and then meet, compare, assess and discuss discrepancies. From that point, every 10th investigation will be double coded, recorded and discussed.

#### Data analysis

The resulting data will be subjected to descriptive statistical analysis to identify patterns and trends and compare results between incident types and investigation methods. The outcome of stage 2 will be new evidence of the quality and the extent to which current investigation methods and models in Australia is consistent with current best practice principles.

### Stage 3—document review

Publicly available board, executive and departmental patient safety plans and committee minutes will be analysed to inform the context of current investigation practices in preparation to further explore these in the stage 4 focus groups and interviews.

### Stage 4—focus groups and interviews

#### Purpose

The focus groups/interviews aim to understand stakeholders’ expectations, experiences and perceptions of investigations, their strengths and weaknesses and current practices and innovations and use this information to codesign best practice guidance. This stage will gather information relating to all three research questions.

#### Setting

The study setting is health services within the four partner jurisdictions.

#### Participants

For health service staff involved in investigations, we will hold 2–3 focus groups per research question in each of NSW, Queensland and Victoria, and 1–2 in ACT. We will also hold 5–8 consumer interviews in each of the four jurisdictions and complete up to 15 semistructured interviews with staff in health services utilising innovative practices in the investigation process. Our sample size will be between 120 and 255 health service staff and 20–32 consumers in total.

##### Staff focus group recruitment

For the staff focus groups, stratified convenience sampling will be used to ensure representation of people who undertake or commission investigations. No more than three staff per health service will participate to enable us to collect a breadth of information across the health services.

To recruit participants, jurisdiction-based patient safety and quality leads will email health service patient safety and quality leads, who will distribute the invite to relevant staff. After participants complete the consent form and demographic information, the research team will contact the participants to invite them to an online focus group. The focus groups will be conducted over 12 weeks and continue until the target sample number is reached or data saturation occurs.

##### Innovation interview recruitment

For the innovation sample group, we will select a convenience sample using the networks of our chief investigators and our partner organisations. These people will be selected based on either their experience in investigations or their use of innovative investigation methods (eg, processes that facilitate consumer involvement on investigation teams). Once identified as a potential participant, they will receive an email invitation to participate from our network connections.

##### Consumer interview recruitment

Consumers (patients who have been impacted by an adverse event) will be identified and invited to participate in two ways: by jurisdiction-based quality and safety leads or health service quality and safety leads, and from consumer healthcare organisations, both national (eg, Consumers Health Forum) and state based. Those consumers who express interest will then receive a formal email invitation, with an electronic link to the participant information and consent form, which will be hosted at Macquarie University on RedCap.^44 45^ RedCap (Research Electronic Data Capture) is a secure, web-based software platform designed to support data capture for research studies.

#### Data collection

Initial demographic information will be collected for all participants. Focus groups and interviews will be audio-recorded. Focus groups will be up to an hour in length, and interviews will be up to 30 min in duration. All focus groups will be with people in the same jurisdiction.

Staff focus groups will explore with participants how they make decisions about what to investigate, what is the ideal criteria for determining this decision and what tools are needed to support the decision-making process, adverse event investigation and the development of recommendations. Focus group discussions will canvas views from participants about how organisations can improve their investigative responses to achieve more effective, sustainable change from recommendations. Semistructured interviews with consumers will focus on understanding what patients and their families need from the investigative process and how those needs can be best met. Focus group and interview questions are provided in online supplemental material 2-9. The questions were informed by safety science^21 23 36 46–53^ and implementation science (eg, the Consolidated Framework for Implementation Research^54 55^) literature and the expertise of our research team.

10.1136/bmjopen-2024-085854.supp2 Supplementary data

#### Data processing and analysis

Audio recordings will be transcribed verbatim by the research team and once checked against the audio recording, imported into NVivo V.20 software for data organisation. The transcripts will be inductively analysed using thematic analysis techniques.^56^ Two analysts working together will conduct the coding to ensure that the process is rigorous, to discuss themes and their concomitant categories, and to arrive at a consensus opinion if any variance in agreement occurs. Finally, we will draw on further literature to find useful analytic concepts to make meaning of the patterns we identify in the data.

### Stage 5—develop best practice guidance

The aim of stage 5 is to develop best practice guidance for investigations into adverse events. The best practice guidance will be informed by the findings from the previous stages and will align with the study’s three research questions. The guidance will be developed using codesign, seeking consumers and health service staff experiences^57^ to both define the problem and design the solution.^58^ Stage 4 is focused on defining the *problem,* while stage 5 is focused on designing the *solutions* including an understanding of how to measure the impact of these.

Data from the previous stages will be analysed and then triangulated. Triangulation will be undertaken across both data (multiple health services and jurisdictions) and methods (eg, policy documents, experiences/perceptions of staff and consumers and investigation quality).^59^ The results will be presented to health service staff, jurisdictional leadership and consumers via the project governance processes and then principles of best practice models and decision-making criteria for learning from adverse events will be codrafted and codeveloped with the stakeholders. Small codesign groups will be then formed in each of the four jurisdictions. Each group will comprise a mix of health service staff and consumers. Iterative design workshops will be held and their content will depend on the analysis of data collected in previous stages. Each group will be facilitated by a member of the research team. These groups will meet in person, in order to facilitate an interactive discussion and shared decision-making process.^57 58^ There will be a set of two design workshops:

- The first will develop shared agreement of the priority areas, and then identify the tools/guidance that may be suitable for these problems. The research team will present an overview of existing strategies. The design group will brainstorm how the priority areas may be addressed, whether existing tools can be used or whether a novel approach is required.
- The second will adapt or further develop the identified tools/guidance and potentially plan feasibility testing.

After the design groups, we will hold focus groups to feedback what has been heard about tools and guidance and seek further input on the outcomes with a wider group. These findings will be presented to policymakers, safety and quality staff, clinicians and patient representatives in seven (two per state and one in the ACT) highly interactive workshops to challenge and refine the models. Safety experts from other high-risk industries (eg, transport using (author) PS’ extensive networks) will be invited, and we will integrate their knowledge and principles where applicable. Scenarios of patient safety adverse events will be used to inform and frame the workshops and to allow simulated small scale testing to occur.^60^ Workshops may be held for different contexts, such as jurisdiction, health service size/rurality and specialty (eg, mental health). The outcome of this stage will be best practice for health services to respond to adverse events.

### Stage 6—feasibility testing

Feasibility testing of the best practice guidance for adverse events response will be conducted in three health services in each of the three states (including one rural each), and one in ACT. Within each of the 10 health services, we will conduct, feasibility, reliability and validity tests on the best practice guidance when responding to five adverse events (n=50 adverse events) over 1 year. We will conduct tests on best practice using a hybrid type 1 feasibility design.^61^ Reliability and validity testing will be informed by safety science methods.^60^ Data collection tools will be staff surveys and audits against adherence to the best practice guidance developed in stage 5. Iterative changes to the best practice guidance will be undertaken based on our findings. The outcome of this stage will be robust feasibility-tested best practice guidance for health services to respond when patients are seriously harmed.

#### Ensuring study quality

The project will be judged by the utility and use of project outcomes by practitioners to spread learning across the system, to engender change in clinical practice, and ensure the tools are consumer-focused. While the research project team conduct the day-to-day work, all chief investigators and associate investigators, consumer representatives and project partners comprise a Steering Committee that meets every 2 months and is responsible for governance, including the overall project direction, monitoring quality and high-level decision-making to ensure utility, feasibility and quality. An international advisory group meets two times a year to provide an international perspective.

## Patient and public involvement

This research study incorporates, as an integral component of its work, a policy and advisory group with consumer representatives involved in its design and conduct.

## Ethics and dissemination

### Ethics

The study will be conducted in compliance with all stipulations of this protocol, the conditions of ethics committee approval, the NHMRC National Statement on Ethical Conduct in Human Research (2007), and the Note for Guidance on Good Clinical Practice (CPMP/ICH-135/95)

The study has received ethics approval from the Northern Sydney Local Health District Human Research Ethics Committee.

### Dissemination

The research findings will be published in international and national journals and disseminated through conferences and presentations to the various stakeholder groups, including consumers, healthcare professionals, policymakers, healthcare organisations and facilities and researchers. The findings from the project will be presented in various forms. We will write peer-reviewed papers targeting publication in relevant international journals, with the intention of publishing the results widely. Conference presentations and presentations to stakeholders' groups, including those involving policymakers, will be pursued. We will carry out a follow-up study to determine the impact of this work on reducing preventable harm.

The findings will be incorporated into best practice guidance of the partners’ policies related to responding to adverse events. The associated tools and guidance will be shared online as well as within training workshops.
