# Supplementary material for: Improving health system responses when patients are harmed: a protocol for a multistage mixed-methods study
Source: BMJ Open. 2024 Jul 5;14(7):e085854. doi: 10.1136/bmjopen-2024-085854 (PMC11227800; doi:10.1136/bmjopen-2024-085854)
Supplement: Supplementary data [file bmjopen-2024-085854supp001.pdf]

Supplementary File 1: Fields for Scoring investigations

Demographic data

Information Required	Field
**1.** Record/investigation number (original from jurisdiction)	Free Text
**2.** The investigation method	Drop Down Menu:<br>• RCA<br>• RCA2<br>• London Protocol<br>• Accimaps/SEIPS<br>• Cluster Map<br>• HEAPS<br>• Other
**1.** State/ Territory	Drop Down:<br>• Australian Capital Territory (ACT)<br>• New South Wales<br>• Queensland<br>• Victoria
**2.** Health Service or Location	Free Text
**3.** Hospital Peer Grouping	**Peer Grouping**<br>Principal referral hospital<br>Public acute group A hospital<br>Public acute group B hospital<br>Public acute group C hospital<br>Public actue group D hospital

	Very small hospital<br>Specialised hospitals group<br>Women's and children's hospitals<br>Psychiatric hospitals<br>Drug and alcohol hospitals<br>Same day hospitals<br>Other Acute Specialised<br>Sub-acute and non-acute hospitals<br>Outpatient hospitals<br>Unpeered hospital<br>Early Parenting Centre<br>Unknown<br>Insufficient information to assess
**4.** Facility Rurality	Drop down:<br>• Major City<br>• Inner Regional<br>• Outer Regional<br>• Remote<br>• Very Remote<br>• Insufficient information to assess
**5.** Admission Type	Elective<br>Emergency<br>Unknown<br>NA
**6.** Admitting Specialty (1)	**Lookup Medical Specialty**<br>**Medical Specialties**<br>Anaesthetics

	Cardiology<br>Critical care<br>Emergency<br>General Ward<br>Imaging<br>Intensive<br>Laboratory<br>Medicine (non-surgical)<br>Mental health/Psychiatry<br>Obstetrics/Gynaecology<br>Oncology<br>Orthopaedics<br>Paediatrics<br>Pharmacy<br>Perioperative<br>Radiology<br>Recovery<br>Surgery<br>Other (Please Specify)	
Medical Specialty related to the incident (2)	**Lookup Medical Specialty**<br>**Medical Specialties**<br>Anaesthetics<br>Cardiology<br>Critical care<br>Emergency<br>General Ward<br>Imaging	

	Intensive<br>Laboratory<br>Medicine (non-surgical)<br>Mental health/Psychiatry<br>Obstetrics/Gynaecology<br>Oncology<br>Orthopaedics<br>Paediatrics<br>Pharmacy<br>Perioperative<br>Radiology<br>Recovery<br>Surgery<br>Other (Please Specify)
Medical Specialty related to the incident (3)	**Lookup Medical Specialty**<br>**Medical Specialties**<br>Anaesthetics<br>Cardiology<br>Critical care<br>Emergency<br>General Ward<br>Imaging<br>Intensive<br>Laboratory<br>Medicine (non-surgical)<br>Mental health/Psychiatry<br>Obstetrics/Gynaecology

	Oncology<br>Orthopaedics<br>Paediatrics<br>Pharmacy<br>Perioperative<br>Radiology<br>Recovery<br>Surgery<br>Other (Please Specify)
**7.** Age of patient involved	• 0-4<br>• 5-9<br>• 10-14<br>• 15-19<br>• 20-24<br>• 25-29<br>• 30-34<br>• 35-39<br>• 40-44<br>• 45-50<br>• 50-54<br>• 55-59<br>• 60-64<br>• 65-69<br>• 70-74<br>• 75-79<br>• 80-84<br>• 85-89<br>• 90-94<br>• 95-99<br>• 100+

**8.** Gender of patient involved	• Male<br>• Female<br>• Non-binary/other
**9.** Incident location	• Operating theatre<br>• General ward/patient's room<br>• Emergency department<br>• Intensive Care Unit<br>• Birthing suite, labour room<br>• Diagnostic procedures (e.g., CT or MRI scan, X-ray, imaging)<br>• Day procedure, treatment room<br>• Mental health, psychiatric unit<br>• Transfer between hospitals or units<br>• Outpatient clinic<br>• Neonatal or paediatric ICU<br>• High Dependence Unit<br>• Long-term acute care, hospice<br>• Coronary care or acute care unit<br>• Nursery<br>• Multiple<br>• Not known<br>• Other
	Other: Free Text
Notifiable Event	YES/NO<br>Reason:
Sentinel Event	Yes/No<br>If yes:<br>**Surgical or other procedure:**<br>**1.** Surgery or other invasive procedure performed on wrong site resulting in serious harm or death<br>**2.** Surgery or other invasive procedure performed on the wrong patient resulting in serious harm or death<br>**3.** Wrong surgical or other invasive procedure performed on a patient resulting in serious harm or death

	**4.** Unintended retention of a foreign object in a patient after surgery or other invasive procedure resulting in serious harm or death<br>**5.** Haemolytic blood transfusion reaction resulting from ABO incompatibility resulting in serious harm or death<br>**Mental Health**<br>**6.** Suspected suicide of a patient within an acute psychiatric unit or acute psychiatric ward<br>**Medication**<br>**7.** Medication error resulting in serious harm or death<br>**Care Management**<br>**8.** Use of physical or mechanical restraint resulting in serious harm or death<br>**9.** Discharge or release of a child to an unauthorised person<br>**10.** Use of an incorrectly positioned oro- or naso-gastric tube resulting in serious harm or death<br>**11.** Other – Catastrophic<br><br>Note: Sentinel Events came from : Australian Sentinel Events List (version 2) Specifications 2020 - australian_sentinel_events_list_version_2_specifications_april_2020.pdf (safetyandquality.gov.au)
Is this multi-agency?	Yes/No
Length of Main report in pages	Free Text: # (number)

Modified International Classification for Patient Safety (ICPS)

The underlying incident involved in Investigation Report will be classified using the World Health Organisation's International Classification for Patient Safety (ICPS)[10] incident type domain, for example, clinical process/procedure, clinical administration, and problems associated with nutrition. These codes can classify how care was not delivered optimally, such as problems with assessment or diagnoses, observations not monitored, or escalated when abnormal, and treatment not indicated.

**Assigned Study ID**	
**Description of Incident**	Free Text
Incident Type 1	**Lookup Incident types**

**Incident Types**
Clinical Administration
Clinical Process/Procedure
Documentation
Healthcare Associated Infection
Medication/IV Fluids
Blood/Blood Products
Nutrition
Oxygen/Gas/Vapour
Medical Device/Equipment
Behaviour
Patient accidents
Falls
Infrastructure/Buildings/Fixtures
Resources/Organizational Management

What went wrong 1	**Lookup What went wrong**	
	**What went wrong**	
	CA_Handover	
	CA_Appointment	
	CA_Waiting List	
	CA_Referral/Consultation	
	CA_Admission	
	CA_Discharge	
	CA_Transfer of Care	
	CA_patient Identification	
	CA_Consent	
	CPP_Screening/Prevention/Routine Check-up	
	CPP_Diagnosis/Assessment	
	CPP_Procedure/Treatment/Intervention	
	CPP_General Care/Management	
	CPP_Tests/Investigations	
	CPP_Specimens/Results	
	CPP_Detention/Restraint	
	CPP_Clinical Orders	
	CPP_Deterioration	
	DocInv_Orders/Requests	
	DocInv_Charts/Medical Records/Assessements/Consultations	
	DocInv_Check Lists	
	DocInv_Forms/Certificates	
	DocInv_Instructions/Information/Policies/Procedures/Guidelines	
	DocInv_Labels/Stickers/Identification Bands/Cards	
	DocInv_Letters/E-Mails/records of Communication	

	DocInv_Investigations/incident reports
	DocInv_Reports/Results/Images
	HAISite_bloodstream
	HAISite_Surgical Site
	HAISite_Abscess
	HAISite_Respiratory
	HAISite_Intravascular Cannulae
	HAISite_Infected Prosthesis/Site
	HAISite_Urinary Drain/Tube
	HAISite_UTI
	HAISite_Cellulitis
	HAISite_Conjunctivitis
	HAISite_VRE
	HAISite_MRSA
	HAISite_Gastroenteritis
	HAISite_Wound
	Med_Prescribing
	Med_Preparation/Dispensing
	Med_Presentation/Packaging
	Med_Delivery
	Med_Administration
	Med_Supply/Ordering
	Med_Storage
	Med_Monitoring
	Bl_Pre-Transfusion Testing
	Bl_Prescribing
	Bl_Preparation/Dispensing

	Bl_Delivery	
	Bl_Administration	
	Bl_Storage	
	Bl_Monitoring	
	Bl_Presentation/Packaging	
	Bl_Supply/Ordering	
	Nut_Prescribing/Requesting	
	Nut_preparation/Manufacturing/Cooking	
	Nut_Supply/Ordering	
	Nut_Presentation	
	Nut_Dispensing/Allocation	
	Nut_Delivery	
	Nut_Administration	
	Nut_Storage	
	O2_Cylinder Labelling/Color Coding/PIN Indexing	
	O2_Prescription	
	O2_Administration	
	O2_Delivery	
	O2_Supply/Ordering	
	O2_Storage	
	Infra_Signage	
	Device_medical device / equipment	
	Beh staff/pt_Behaviour	
	Acc_Pt accident	
	Infra_Infrastructure / building fixture	
	Resources / organisational management	
	Fall__involving_Cot	

	Fall__involving_Bed<br>Fall__involving_Chair<br>Fall__involving_Stretcher<br>Fall__involving_Toilet<br>Fall__involving_Therapeutic Equipment<br>Fall__involving_Stairs/Steps<br>Fall__involving_Being Carried/Supported by Another Individual
Why it went wrong 1	**Lookup Why it went wrong**<br>**Why it went wrong**<br>CA_Not Performed when Indicated<br>CA_Incomplete/Inadequate<br>CA_Unavailable<br>CA_Wrong Patient<br>CA_Wrong Process/Service<br>CPP_Not Performed when Indicated<br>CPP_Incomplete/Inadequate<br>CPP_Unavailable<br>CPP_Wrong Patient<br>CPP_Wrong Process/Treatment/Procedure<br>CPP_Wrong Body Part/Side/Site<br>CPP_Not followed<br>CPP_Actions not aligned with policy/procedure<br>CPP_Actions not aligned with care plan<br>CPP_Not done<br>CPP_Not detected<br>DocP_Document Missing or Unavailable<br>DocP_Delay in Accessing Document

	DocP_Document for Wrong Patient or Wrong Document	
	DocP_Absent/no information in Document	
	DocP_not done/actioned	
	DocP_Multiple versions	
	DocP_Unclear/Ambiguous/Illegible/Incomplete/Information in Document	
	HAIType_Bacteria	
	HAIType_Virus	
	HAIType_Fungus	
	HAIType_Parasite	
	HAIType_Protozoa	
	HAIType_Rickettsia	
	HAIType_Prion	
	Med_Not given when indicated	
	Med_Not indicated	
	Med_Not checked	
	Med_Wrong Patient	
	Med_Wrong Drug	
	Med_Wrong Dose/Strength of Frequency	
	Med_Wrong Formulation or Presentation	
	Med_Wrong Route	
	Med_Wrong Quantity	
	Med_Wrong Dispensing Label/Instruction	
	Med_Contraindication	
	Med_Wrong Storage	
	Bl_Wrong Patient	
	Bl_Wrong Blood/Blood Product	
	Bl_Wrong Dose/Frequency	

	Bl_Wrong Quantity	
	Bl_Wrong Dispensing Label/Instruction	
	Bl_Contraindicated	
	Bl_Wrong Storage	
	Bl_Omitted Blood/Blood Product or Dose	
	Bl_Expired Blood/Blood Product	
	Nut_Wrong Patient	
	Nut_Wrong Diet	
	Nut_Wrong Quantity	
	Nut_Wrong Frequency	
	Nut_Wrong Consistency	
	Nut_Wrong Storage	
	Nut_Not given	
	O2_Wrong patient	
	O2_Wrong Gas/Vapor	
	O2_Wrong Rate/Flow/Concentration	
	O2_Wrong Delivery Mode	
	O2_Contraindication	
	Device_Poor Presentation/Packaging	
	Device_Lack of Availability	
	Device_Innapropriate for Task	
	Device_Unclean/Unsterile	
	Device_Failure/Malfunction	
	Device_Dislodgement/Misconnection/Removal	
	Device_Use Error	
	BehStaff_Noncompliant/Uncooperative/Obstructive	
	BehStaff_Inconsiderate/Rude/Hostile/Inappropriate	

	BehStaff_Risky/Reckless/Dangerous
	BehStaff_Problem with Substance Use/Abuse
	BehStaff_Harassment
	BehStaff_Discrimination/Prejudice
	BehStaff_Wandering/Absconding
	BehStaff_Intended Self Harm/Suicide
	BehStaff_Verbal Aggression
	BehPatient_Noncompliant/Uncooperative/Obstructive
	BehPatient_Inconsiderate/Rude/Hostile/Inappropriate
	BehPatient_Risky/Reckless/Dangerous
	BehPatient_Problem with Substance Use/Abuse
	BehPatient_Harassment
	BehPatient_Discrimination/Prejudice
	BehPatient_Wandering/Absconding
	BehPatient_Intended Self Harm/Suicide
	BehPatient_Verbal Aggression
	Acc_Contact with Object or Animal
	Acc_Contact with Person
	Acc_Crushing
	Acc_Abrading/Rubbing
	Acc_Scratching/Cutting/Tearing/Severing
	Acc_Puncturing/Stabbing
	Acc_Biting/Stinging/Invenomating
	Acc_Other Specified Piercing/Penetrating Force
	Patient Accidents - Other Mechanical Force
	Acc_Struck by Explosive Blast
	Acc_contact with machinery

	Acc_Excessive Heat/Fire<br>Acc_Excessive Cooling/Freezing<br>Acc_Mechanical Threat to Breathing<br>Acc_Drowning/Near Drowning<br>Acc_Confinement to Oxygen-Deficient Place<br>Acc_Poisoning by Chemical or Other Substance<br>Acc_Corrosion by Chemical or Other Substance<br>Acc_Exposure to Electricity/Radiation<br>Acc_Exposure to Sound/Vibration<br>Acc_Exposure to Air Pressure<br>Acc_Exposure to Low Gravity<br>Acc_Exposure to (effect of) Weather, Natural Disaster, or Other Force of Nature<br>Fall_Trip/Stumble<br>Fall_Slip<br>Fall_Collapse<br>Fall_Loss of Balance<br>Infra_Non-Existent/Inadequate<br>Infra_Damaged/Faulty/Worn<br>Reso_Staff not avaliable<br>Reso_Knowlegde and skills<br>Reso_Matchinng of workload Management<br>Reso_Bed/Service Availability/Adequacy<br>Reso_Human Resource/Staff Availability/Adequacy<br>Reso_Organization of Teams/People<br>Reso_Protocols/Policy/Procedure/Guideline Availability/Adequacy
Incident Type 2	As Incident type 1

What went wrong 2	As 1
Why it went wrong 2	As 1
Incident type 3	As incident type 1
What went wrong 3	As 1
Why it went wrong 3	As 1
Other comments	Free text

Contributing Factors

Assigned Study ID	
Number of contributing factors	Free Text: # (number)
Contributing Factor ID	Free Text (Number)
Contributing Factor Text	Free Text
SEIPS Parent Category	**Lookup SEIPS levels**<br>**SEIPS Levels**<br>External Influences<br>Organisation of work factors<br>Person factors<br>Task factors<br>Tool and technology<br>Physical environment

SEIPS Category	**Lookup SEIPS categories**<br>**SEIPS categories**<br>Societal influence (External influences)<br>Macro-economic funding influences (External influences)<br>Policy regulations & targets (External influences)<br>Organisational Structure and governance (Organisational Factors)<br>Culture Leadership and communication (Organisational Factors)<br>Policy and Procedures (Organisational Factors)<br>Workforce management (Organisational Factors)<br>Competency management systems (Organisational Factors)<br>Administration systems (Organisational Factors)<br>Non/Clinical central services (Organisational Factors)<br>Staff factors (Work - Tasks, People and actions)<br>Care team factors (Work - Tasks, People and actions)<br>Patient/Family and Carers factors (Work - Tasks, People and actions)<br>Task design factors (Work - Tasks, People and actions)<br>Workload factors (Work - Tasks, People and actions)<br>Local Procedures Job aids (Equipment and surroundings)<br>Equipment (Equipment and surroundings)<br>Workspace (Equipment and surroundings)<br>Other (Please Specify)
Potential Contributing factors (implicated not further considered in report)	Free text

Recommendations

Assigned Study ID	
Number of recommendations	Free Text: # (number)
Recommendation ID	Free Text (Number)
Recommendation Text	Free Text
Recommendation Type/Strength	**Lookup Rec Type (Strength)**

**Rec Type**
Architectural/physical plant changes (including checking for hanging points) (Strong)
Standardize on equipment or process including benchmarking against other organizations (Strong)
New devices with usability testing (Strong)
Engineering control (forcing function) (Strong)
Tangible involvement by leadership (including establishing clinical governance) (Strong)
Simplify Process (Strong)
Policy/guidelines/ documentation etc. review/enhancement (Medium)
Audit undertaken (Medium)
Enhanced documentation, communication (Medium)
Review rostering/appropriateness of staff mix (Medium)
Re-evaluate use/approriatness of equipment (Medium)
Checklist/cognitive aids (Medium)
Standardized communication tools (Medium)
Software enhancements, modifiction (Medium)

	Education using simulatio based training, with periodic refresher sessions/observations (Medium)<br>Increase in staffing/decrease in workload (Medium)<br>Implement a new team eg MET Team initiated (excludes high level teams such as formed clinical governance) (Medium)<br>Eliminate/reduce distractions (Medium)<br>Redundancy (Medium)<br>Eliminate look- and sound alikes (Medium)<br>Training and Education (including counselling) (Weak)<br>New procedure/memorandum/policy (Weak)<br>Formal discussion/taken to meeting (Weak)<br>Informing/Notifying/Warning (Weak)<br>Double Checks (Weak)<br>Warning (Weak)<br>Other (Please Specify)<br>No recommendations
Recommendation General Comments	Free text

IGZ Tool

This assesses adherence to guidance for patient safety investigation methods (e.g., investigation process and team, incident reconstruction, analysis, conclusion, recommendations, aftercare, and Board responses). A set of questions are applied to judge the quality of an investigation report. The questions are used to judge the quality of event analysis report. Points can be scored with each question. Sometimes a question is irrelevant, for example, 'Was external expertise consulted?', when external expertise would not add anything of value to the analysis. The total amount of points is divided by the total amount of relevant questions, leading to a percentage. This percentage is the overall score of the sentinel event analysis report. Scores for all reports can be aggregated to provide overall average score for a facility or healthcare service that produces reports.

IGZ Question	Answer	Provide Details
1. How soon after the event was identified did the investigation start?	Free Text	NA
2. Is the investigating committee Multidisciplinary?	Yes/No /NA/Insufficient Information	NA
3. Were any members of the investigating committee involved in the incident?	Yes/No /NA/Insufficient Information	NA
4. Was input sought from all personnel directly involved	Yes/No /NA/Insufficient Information	NA
5. Was input sought from other staff with knowledge about the care process?	Yes/No /NA/Insufficient Information	NA
6. Was input sought from the patient/relatives?	Yes/No /NA/Insufficient Information	Free text
7. Does the description of the event give a complete picture of the relevant "scenes"?	**Dropdown:**<br>Brief (basic info insufficient for picturing the event);<br>Moderate (good overview by lacking in some details for full understanding);<br>Extensive (allowing full understanding and reconstruction of event);Insufficient information to assess	Free text
8. Has the question "why" been asked extensively enough to analyse the underlying cause and effect?	Yes/Partially/No /NA/Insufficient Information	Free text

9. Have the investigators searched relevant scientific literature?	Yes/No /NA	Free text
10. Does the report state whether the applicable guidelines/protocols were followed?	Yes/Partially/No /NA/Insufficient Information	Free text
11. Was external expertise consulted?	Yes/No /NA/Insufficient Information	Free text
12. Does the report state whether the medical indication for the provided care was correct?	Yes/No /NA/Insufficient Information	Free text
13. Do the contributing factors it the reconstruction and analysis?	Yes/Partially/No /NA/Insufficient Information	Free text
14. Are contributing factors, not under the control of the hospital, considered and/or identified?	**Dropdown:**<br>• Yes (factors external to hospital are considered and identified as contributory where appropriate)<br>• Partial (some factors beyond the hospital are mentioned but not identifed as contributory)<br>• No<br>• NA<br>• Insufficient information to assess	Free text
15. Does the report document recommendations for improving processes and systems?	Yes/Partially/No /NA/Insufficient Information	Free text
16. Do these corrective actions address the identified root causes?	Yes/Partially/No /NA/Insufficient Information	Free text
17. Have the corrective actions been formalised? (e.g., Specific, Measurable, Attainable, Realistic and Time-Sensitive (SMART)	Yes/Partially/No /NA/Insufficient Information	Free text
18. Does the hospital have an evaluation plan to determine if the recommendations are implemented?	Yes/Partially/No /NA/Insufficient Information	Free text
19. Will the impact of the recommendations be evaluated?	Yes/Partially/No /NA/Insufficient Information	Free text

**Aftercare**		
20. Is the aftercare for the patient/relatives described?	Yes/Partially/No /NA/Insufficient Information	Free text
21. Is the aftercare for the professionals involved described?	Yes/Partially/No /NA/Insufficient Information	Free text
22. Has the report been shared with the patient/relatives?	Yes/No /NA/Insufficient Information	Free text
23. Is the reaction of hospital board adequate?	Yes/No /NA/Insufficient Information	Free text
Any other notes	Free text	NA

Learning Response Review and Improvement Tool

The tool from NHS Education for Scotland studies[34, 35] critically reviews written reports. The tool was developed to (1) identify potential areas for improvement in existing reports to feed back to organisations; (2) Inform future learning response training and report writing practices; (3) Act as a potential quality assurance process for monitoring, evaluating and improving the standard of organisational learning response reports.

Area of Review (descriptor)	Rating Score (Dropdown)	Comments / examples of text quotes
**1. People affected by incidents are meaningfully engaged and involved**<br>The report demonstrates evidence that all those affected by the incident such as staff, patients, families and carers have been actively listened to and emotionally supported where required (i.e., interviews and perspectives of those affected are included in the report).	• Good evidence<br>• Some/Partial Evidence<br>• No evidence<br>• NA<br>• Insufficient information to assess	Free Text
**2. The systems approach is applied**<br>The report demonstrates consideration of system-based performance influencing factors (e.g., task complexity, technology, work procedures, workplace design, information transfer, clinical condition of patient, stress, fatigue, culture, leadership/ management, policy/regulation) and how these interacted to contribute to the incident in question.	• Good evidence<br>• Some/Partial Evidence<br>• No evidence<br>• NA<br>• Insufficient information to assess	Free Text
**3. Human Error' is considered as a symptom of a system problem**<br>'Human error' or similar (e.g., nurse error, medical error, loss of situation awareness) is not concluded to be the cause' of the incident. Instead, multiple contributory factors which influenced the event are explored.	• Good evidence<br>• Some/Partial Evidence<br>• No evidence<br>• NA<br>• Insufficient information to assess	Free Text
**4. Blame language is avoided**<br>Language does NOT directly or indirectly infer blame of individuals, teams, departments, or organisations and/or focus on human failure (i.e., the nurse failed to follow policy; the doctor lost situation awareness).	• Good evidence<br>• Some/Partial Evidence<br>• No evidence<br>• NA<br>• Insufficient information to assess	Free Text

**5. Local rationality is considered**<br>The report clearly explains why the decisions and actions taken by individuals involved felt right at the time (i.e., the situation and context faced by those individuals is explored and described).	• Good evidence<br>• Some/Partial Evidence<br>• No evidence<br>• NA<br>• Insufficient information to assess	Free Text
**6. Counterfactual reasoning is avoided**<br>The report focuses on what happened and understanding why and NOT what people, departments or organisations 'could' or 'should' have done during or before the incident.	• Good evidence<br>• Some/Partial Evidence<br>• No evidence<br>• NA<br>• Insufficient information to assess	Free Text
**7. Safety actions/recommendations are effective** Safety actions/recommendations proposed:<br>• have been developed collaboratively with relevant staff/stakeholders and with consideration of wider organisation priorities and improvement work<br>• focus on system elements (IT, equipment, care processes/pathways) not individuals<br>• are specific, robust and actionable i.e., they don't add to 'safety clutter'<br>• are accompanied by a plan to monitor progress over time<br>• are demonstrably linked to the evidence and findings in the report	• Good evidence<br>• Some/Partial Evidence<br>• No evidence<br>• NA<br>• Insufficient information to assess	Free Text
**8. The written report is clear, easy to read and anonymised**<br>The report is concise, written in plain English, uses inclusive language and anonymised i.e., it is written to 'inform rather than impress	• Good<br>• Average<br>• Poor<br>• NA<br>• Insufficient information to assess	Free Text
**General comments:** Is there anything else that can be improved or content that you thought worked well and should be used in other reports	Free Text	
