# Supplementary material for: Improving health system responses when patients are harmed: a protocol for a multistage mixed-methods study
Source: BMJ Open. 2024 Jul 5;14(7):e085854. doi: 10.1136/bmjopen-2024-085854 (PMC11227800; doi:10.1136/bmjopen-2024-085854)
Supplement: Supplementary data [file bmjopen-2024-085854supp002.pdf]

Supplementary Material

Contents

Supplementary File 2: Stage 4 Research Question 1 Focus Group Schedule Health Service Staff 2

Supplementary File 3: Stage 4 Research Question 2 Focus Group Schedule Health Service Staff 4

Supplementary File 4: Stage 4 Research Question 3 Focus Group Schedule Health Service Staff 5

Supplementary File 5: Stage 4 Interview Schedule Research Question 1-3 Consumers 6

Supplementary File 6: Interview Schedule Health Service Staff Innovation 7

Supplementary File 7: Stage 4 Potential NSW Specific Questions 8

Supplementary File 8: Stage 4 Potential Victoria Specific Questions 9

Supplementary File 9: Stage 5 Follow up focus group 11

Hibbert PD, *et al. BMJ Open* 2024; 14:e085854. doi: 10.1136/bmjopen-2024-085854

Supplementary File 2: Stage 4 Research Question 1 Focus Group Schedule Health Service Staff

Research Question: How should we decide what to investigate, and to what level, to maximise learning to improve patient safety – making best use of the limited resources available?

**Focus Group Questions**
1. How do you decide now what to investigate and to what level? Aside from the official guidelines, what (in practice) influences this decision and why? (how could we do more with less?)
2. What structures do you have in place to decide? What skills and representation do you use?
3. How might novel approached (RJC, Swarm huddle, Rapid Incident Review Meeting (RIRM) etc) change how level of incidents are determined?
4. How are current practices working - In terms of getting the most value from the investigation process? Do you have any tools that you use to decide?
5. IF there were no rules, what would you stop doing? what would you keep doing? How might this change the effectiveness and value of investigations or alter the use of resources?
6. How would you prioritise the 12 criteria from the Grey literature review? – E.g., should severity be the only criteria? if not what else is important and in what order?
7. If we were to develop a tool to help people with triage – who would use it and what features should it have to support each of those user groups?
8. Do you routinely use other methods of detecting safety incidents? If so, what other methods are in use and how does it compare to self-report?
9. How does the Organisations safety and quality system and strategy prioritise resources for investigations and recommendations? (What % of resources are focused on investigating and responding to recs and what % are trying to implement solutions to known problems? Is there a strategy to look at high level problems based on multi-incident analysis?
**Research Sources (Literature):** • Vincent C, Carthey J, Macrae C, et al. Safety analysis over time: seven major changes to adverse event investigation. Implementation Science. 2017;12(1):151.<br>• Turner K, Stapelberg NJ, Sveticic J, et al. Inconvenient truths in suicide prevention: Why a Restorative Just Culture should be implemented alongside a Zero Suicide Framework. Aust N Z J Psychiatry. 2020;54(6):571-81.<br>• Kaur M, De Boer RJ, Oates A, et al. Restorative Just Culture: a Study of the Practical and Economic Effects of Implementing Restorative Justice in an NHS Trust. MATEC Web Conf. 2019;273:01007.<br>• Li J, Boulanger B, Norton J, et al. "SWARMing" to Improve Patient Care: A Novel Approach to Root Cause Analysis. Jt Comm J Qual Patient Saf. 2015;41(11):494-501.<br>• Motuel L, Dodds S, Jones S, et al. Swarm: a quick and efficient response to patient safety incidents. Nursing Times. 2017;12(9):36-8.<br>• Holden RJ, Carayon P, Gurses AP, et al. SEIPS 2.0: a human factors framework for studying and improving the work of healthcare professionals and patients. Ergonomics. 2013;56(11):1669-86.

- University Hospitals Bristol and Weston NHS Foundation Trust. Standard Operating Procedure: Patient safety incident investigation (PSII) rapid incident review meetings (RIRM) Bristol, UK: University Hospitals Bristol and Weston NHS Foundation Trust; 2021. Available from: https://www.uhbw.nhs.uk/assets/1/22-394_patientsafetyincidentinvestigationrapidincidentrev-1_0_redacted.pdf#:~:text=A%20Rapid%20Incident%20Review%20Meeting%20%28RIRM%29%20is%20called,reference%20to%20a%20reported%20incident%20of%20significant%20concern.

Supplementary File 3: Stage 4 Research Question 2 Focus Group Schedule Health Service Staff

Research Question: How can we ensure investigations are of sufficient quality to identify contributing factors and identify effective system Improvements?

**Focus Group Questions**
1. Do you believe the incident investigation method(s) used are effective for prompting change? Are there investigation models or approaches that would work better?
1. How would RJC approach be change the value and effectiveness of investigations? (Psych. first aid, RCAs replaced with facilitated RJC review (forward-looking review of 'the clinical care pathway' not looking back from an incident), involved staff participate in the review, finding solution and sharing the learnings.)
2. How can we learn from what goes right to make sure it happens more of the time in more places?
3. Given the results of the investigation review – what should we do more or less of? what changes are needed to improve the quality of investigations or their recommendations
4. Is the guidance for investigators is adequate? IF not, where is more support needed?
5. What tips and tricks (" cookbook") advice would help panels to get through the investigation process more easily? What further advice or tools would help?
6. How are actions tracked and monitored in each state – is this sufficient to understand the effectiveness and sustainability of recommendation? What else would help (e.g., AI)
7. When do recommendations work? (effective/sustainable) when are they more likely to fail?
8. What big changes are needed to help the health system respond better when patients are harmed (legal, cultural, political, financial, other?)
**Research Sources (Literature):**<br>• Kellogg KM, Hettinger Z, Shah M, et al. Our current approach to root cause analysis: is it contributing to our failure to improve patient safety? BMJ Qual Saf. 2017;26(5):381-7.<br>• Hibbert P, Thomas MJW, Deakin A, et al. Final Report: Sentinel Event Research Project. A report submitted to the Victorian Department of Health and Human Services. Melbourne, Australia: Australian Patient Safety Foundation; 2016.<br>• Vincent C, Carthey J, Macrae C, et al. Safety analysis over time: seven major changes to adverse event investigation. Implementation Science. 2017;12(1):151.<br>• Hibbert P, Schultz T. A review of SA Health and Wellbeing's Safety Learning System. Adelaide, SA: SA Health and Wellbeing; 2020.<br>• Turner K, Stapelberg NJ, Sveticic J, et al. Inconvenient truths in suicide prevention: Why a Restorative Just Culture should be implemented alongside a Zero Suicide Framework. Aust N Z J Psychiatry. 2020;54(6):571-81.

Supplementary File 4: Stage 4 Research Question 3 Focus Group Schedule Health Service Staff

Research Question: Why do the recommended actions from investigations sometime fail to generate systematic, sustainable improvements to patient safety – and how could we improve this for the future?

**Interview/ Focus Group Questions**
1. How are recommendations currently implemented in your health service? Can you describe the process? Who is responsible for this process and what happens in practice?
2. What feedback processes are currently in place in regard to implementation of recommendations? And how is success or failure of implementation of a recommendation measured?
3. What do you think is working well with your recommendation implementation process?
4. If you could change anything in order to make it easier to successfully implement recommendations what would this be?

Hibbert PD, *et al. BMJ Open* 2024; 14:e085854. doi: 10.1136/bmjopen-2024-085854

Supplementary File 5: Stage 4 Interview Schedule Research Question 1-3 Consumers

Interview/ Focus Group Questions
1. When is an investigation required/ not required from a consumer's perspective- What criteria should determine this?
5. When is an investigation needed – what features or processes should be integrated into the process to ensure consumer needs are met?
6. What do consumers need from the investigative process - Is the current process meeting this?
7. How could the needs of Consumers be met – outside of a formal investigation process? What other mechanisms could fulfill this purpose? (E.g., Restorative Just Culture (RJC) process)
8. How would a RJC approach be received by consumers? (interview for consumer perspective of events; and lessons consumers feel need to be learned; then gather any questions that would like answered within the review process. Follow up: Meet to share findings or review, structured interaction, answers to questions raised, feedback on actions taken. Consumer provides post incident feedback)
9. After the investigation has been completed, the health service needs to implement the recommendations. What are the needs of consumers during this time?

Research Sources (Literature):

- The Behavioural Insights Team. Behavioural insights into patient motivation to make a claim for clinical negligence. London, UK: NHS Resolution; 2018.
- Hibbert P, Thomas MJW, Deakin A, et al. Final Report: Sentinel Event Research Project. A report submitted to the Victorian Department of Health and Human Services. Melbourne, Australia: Australian Patient Safety Foundation; 2016.
- Vincent C, Carthey J, Macrae C, et al. Safety analysis over time: seven major changes to adverse event investigation. Implementation Science. 2017;12(1):151.
- Turner K, Stapelberg NJ, Sveticic J, et al. Inconvenient truths in suicide prevention: Why a Restorative Just Culture should be implemented alongside a Zero Suicide Framework. Aust N Z J Psychiatry. 2020;54(6):571-81.

Supplementary File 6: Interview Schedule Health Service Staff Innovation

**Focus Group Questions**
1. What are the main features of the new approach?
2. What are the main benefits and challenges?
3. Has this practice been evaluated?
4. What advice would you have for someone trying to replicate this process?
**Research Sources:**<br>Grey Literature, Previous interviews, and Focus groups

Supplementary File 7: Stage 4 Potential NSW Specific Questions

Consumers in NSW who have been provided with a Dedicated Family Contact during a Serious Adverse Event Investigation

We would like to ask you some questions about the Dedicated Family Contact that was involved in your case.

- Can you describe what the Dedicated Family Contact did in your case?
- What were the benefits to you and your family for Dedicated Family Contact?
- Were there any negative impacts of having a Dedicated Family Contact?
- Were there any barriers to the Dedicated Family Contact helping you?
- Could you make any suggestions to improve the system of providing a Dedicated Family Contact?
- Would you recommend a Dedicated Family Contact for people/families/carers who have been involved in a serious adverse event? Why?

Hibbert PD, *et al. BMJ Open* 2024; 14:e085854. doi: 10.1136/bmjopen-2024-085854

Supplementary File 8: Stage 4 Potential Victoria Specific Questions

Healthcare providers who have been involved in investigations with an independent person and/or a consumer

- How many investigations have you been involved with an independent person and/or a consumer?
- Have you previously been involved with panels without an independent person and/or a consumer?
- Describe your perception of the role of an independent person and/or a consumer on the investigation?
- What tasks within the investigation did they generally undertake?
    - (Prompts: development of timeline, interview questions, undertake interviews, writing up interviews, causation development, recommendation development, report writing, specialised contribution e.g., human factors)
- Comparing investigations with and without an independent person and/or a consumer, what was different?
    - (Prompts: behaviours within the panel, focus of discussions on causes, focus of discussions on recommendations, length of time to undertake investigations, asking questions not raised by other panel members, ensuring consumer voice is not lost, ensuring staff don't get lost in 'clinical detail'.)
- What are the benefits of involvement in investigations of an independent person and/or a consumer?
    - (Prompts: Hearing different ways of doing things, being able to see things clearly without bias, System-thinking, consumer focussed, recommendations more likely to be implemented.)
- What are the weaknesses or risks?
    - (Prompts: Psychological distress, unhealthy unmanaged conflict within the team, focus on blame, nor feeling comfortable speaking up, uncertainty about the role, more difficult to organise or time consuming)
- What are the barriers to including independent person and/or a consumer on an investigation?
- What support do independent person and/or a consumer need from the health service? What support do they need from Safer Care Victoria?
    - (Prompts: training, understanding the investigation methodology, medical jargon, counselling/support)
- What supports/information is provided to independent panel members/consumer, prior to a review commencing.
    - (Prompts: 1:1 meeting, information sheet on what is expected, basic information on the review methodology, a clear outline of role, confidentiality agreement, orientation to electronic systems for information sharing, direct access to medical record)
- What additional skills does a facilitator require if investigations include independent people and/or consumers?
- Prior to your involvement with an independent person/consumer, what information/training/support did you receive regarding their inclusion? Was it adequate?
- Would you recommend investigations include independent people and/or consumers? Why?

Independent person and/or a consumer who have been involved in investigations

- How many investigations have you been involved with?

- Have you previously been involved with panels without an independent person and/or a consumer?
- Describe your perception of the role of an independent person and/or a consumer on the investigation?
- What tasks within the investigation have you generally undertaken?
    - (Prompts: development of timeline, interview questions, undertake interviews, writing up interviews, causation development, recommendation development, report writing, specialised contribution e.g., human factors)
- What are the benefits of involvement in investigations of an independent person and/or a consumer?
    - (Prompts: System-thinking, consumer focussed, recommendations more likely to be implemented.)
- What are the weaknesses or risks?
    - (Prompts: Psychological distress, unhealthy unmanaged conflict within the team, focus on blame, nor feeling comfortable speaking up, uncertainty about the role, more difficult to organise or time consuming)
- What are the barriers to being independent person and/or a consumer on an investigation?

I have some specific questions about the investigations in which you were involved?

- Were you involved in the creation of recommendations?
- After the conclusion of the investigation did you see the final report?
- Did you feel you had sufficient psychological supports in place?
- Did you feel you could speak up?
- Prior to your role as an independent person/consumer, what information/training/support did you receive? Was it adequate?
- What support do independent person and/or a consumer need from the health service? What support do they need from Safer Care Victoria?
    - (Prompts: training, understanding the investigation methodology, medical jargon, counselling/support)
- What additional skills does a facilitator require if investigations include independent people and/or consumers?
- Do you feel you provided a value add to the review? If so in what way?
- Would you recommend investigations include independent people and/or consumers? Why?
- Would you do it again? Why/Why not?

Supplementary File 9: Stage 5 Follow up focus group

**Focus Group Questions**
1. How well does the guidance address the user needs identified? (Including the needs identified in the investigation review)
2. What challenges could you envisage when trying to adopt the recommendations and guidance?
3. Do you see any risks with using the tools?
4. Whilst using the tool with case studies – how useful was the tool?
5. How valuable would a tool like this be in your organisation, how would it change the use of resources, what challenges and benefits could you imagine?
6. Would you be willing to trial the guidance in your organisation and provide feedback
**Research Sources:**<br>User requirements and best practice identified from Strategic Review and Grey literature, international interviews and innovation interviews.

Hibbert PD, *et al. BMJ Open* 2024; 14:e085854. doi: 10.1136/bmjopen-2024-085854
